# Resolving an inconsistency in the estimation of the energy for excitation of cardiac muscle contraction

**DOI:** 10.3389/fphys.2023.1269900

**Published:** 2023-10-03

**Authors:** June-Chiew Han, Toan Pham, Andrew J. Taberner, Denis S. Loiselle, Kenneth Tran

**Affiliations:** ^1^ Auckland Bioengineering Institute, The University of Auckland, Auckland, New Zealand; ^2^ Department of Engineering Science and Biomedical Engineering, The University of Auckland, Auckland, New Zealand; ^3^ Department of Physiology, The University of Auckland, Auckland, New Zealand

**Keywords:** cardiac energetics, activation heat, pressure-volume area, force-length work, calcium handling, end-systolic

## Abstract

In the excitation of muscle contraction, calcium ions interact with transmembrane transporters. This process is accompanied by energy consumption and heat liberation. To quantify this activation energy or heat in the heart or cardiac muscle, two non-pharmacological approaches can be used. In one approach using the “pressure-volume area” concept, the same estimate of activation energy is obtained regardless of the mode of contraction (either isovolumic/isometric or ejecting/shortening). In the other approach, an accurate estimate of activation energy is obtained only when the muscle contracts isometrically. If the contraction involves muscle shortening, then an additional component of heat associated with shortening is liberated, over and above that of activation. The present study thus examines the reconcilability of the two approaches by performing experiments on isolated muscles measuring contractile force and heat output. A framework was devised from the experimental data to allow us to replicate several mechanoenergetics results gleaned from the literature. From these replications, we conclude that the choice of initial muscle length (or ventricular volume) underlies the divergence of the two approaches in the estimation of activation energy when the mode of contraction involves shortening (ejection). At low initial muscle lengths, the heat of shortening is relatively small, which can lead to the misconception that activation energy is contraction mode independent. In fact, because cardiac muscle liberates heat of shortening when allowed to shorten, estimation of activation heat must be performed only under isometric (isovolumic) contractions. We thus recommend caution when estimating activation energy using the “pressure-volume area” concept.

## Introduction

Twitch force produced by cardiac muscle requires energy. That energy is consumed by ATPases that drive cardiac excitation-contraction. That energy is liberated entirely as heat when the muscle contracts isometrically. When the contraction involves shortening, then a portion of the energy is used to do force-length work, with the remaining liberated as heat (Gibbs and Chapman, 1979; Loiselle et al., 2016). The energy that is consumed is recovered by mitochondrial oxidative phosphorylation, almost immediately, with negligible temporal distinction, even at temperature as low as 20°C (Gibbs and Chapman, 1979).

During twitch force production, a portion of the energy is used for cellular Ca^2+^ handling, and is accompanied by release of heat, which is coined ‘activation heat’ (Gibbs et al., 1988; Han et al., 2019b). The heat release comes from i) ATP hydrolysis by transmembrane transporters to remove Ca^2+^ from the cytosol following Ca^2+^-induced Ca^2+^ release, and ii) ATP replenishment from mitochondrial oxidative phosphorylation. The transmembrane transporters involved in this process are the sarcoplasmic reticular Ca^2+^ ATPase, sarcolemmal Ca^2+^ ATPase, and Na^+^-K^+^ ATPase in concert with the Na^+^-Ca^2+^ exchanger.

Using well-designed experimental protocols and maneuvers, the total heat liberated from cardiac contraction can be separated into basal (resting; contraction-independent) heat and suprabasal (contraction-dependent) heat (Gibbs and Loiselle, 2001). Suprabasal heat is further partitioned into activation heat and crossbridge heat which is associated with the actomyosin ATPase and can be further distinguished into isometric heat and shortening heat (Tran et al., 2017).

The measurement of activation heat in cardiac muscle was first made in the 1960s by C. L. Gibbs and others on isolated papillary muscles (Ricchiuti and Gibbs, 1965). This extended his endeavours to measure activation heat in skeletal muscles (Gibbs and Ricchiutti, 1965) that were based on the seminal work of A. V. Hill (Hill, 1949). A. V. Hill first defined activation heat as ‘… *a* ‘*triggered’ reaction setting the muscle in a state in which it can shorten and do work*’ (Hill, 1949). Since the 1970s, pursuant to investigations of muscle excitation-contraction coupling, the definition of activation heat has expanded to ‘*the sum of the thermal accompaniments of the liberation of calcium into the sarcoplasm, its movement to and from the myofibrillar binding sites, and its return to its storage site by an ATP-dependent transport process in the sarcoplasmic reticulum*’ (Homsher et al., 1972). C. L. Gibbs has ascribed activation heat, in a much-abbreviated form, to “*the cost of sarcoreticular and sarcolemmal Ca* ^*+ +*^ *pumping*” (Gibbs, 1987).

For skeletal muscles, an elegant method to measure the magnitude of activation heat involves stretching the muscle preparations beyond their optimal length such that the interaction between the thick and thin filaments vanishes (Homsher et al., 1972). This manoeuvre risks negligible irreversible tissue damage, while allowing separation of the energy cost of the activation mechanism from the energy expenditure by crossbridge cycling. By studying semitendinosus muscles stretched to various lengths beyond the optimal length, twitch heat was found to be linearly related to twitch force, where the *y*-intercept defines the activation heat (Homsher et al., 1972). For cardiac muscle, in contrast, to avoid irreversible tissue damage, activation heat was determined by shortening the muscle until little or no active force was developed on stimulation (Ricchiuti and Gibbs, 1965). In isometrically-contracting ventricular papillary muscles gradually pre-shortened to various lengths below the optimal length, heat was plotted against stress (force per cross-sectional area), where the *y*-intercept of the relation quantifies activation heat (Loiselle and Gibbs, 1979; Gibbs et al., 1988). A much higher *y*-intercept was obtained when papillary muscles were subjected to isotonic contractions (Gibbs et al., 1967; Gibbs and Gibson, 1970b) or to latency quick-release contractions (Gibbs, 1987; Gibbs et al., 1988). Thus, cardiac muscle was thought to produce different magnitudes of activation heat depending upon the mode of contraction.

**FIGURE 1:**
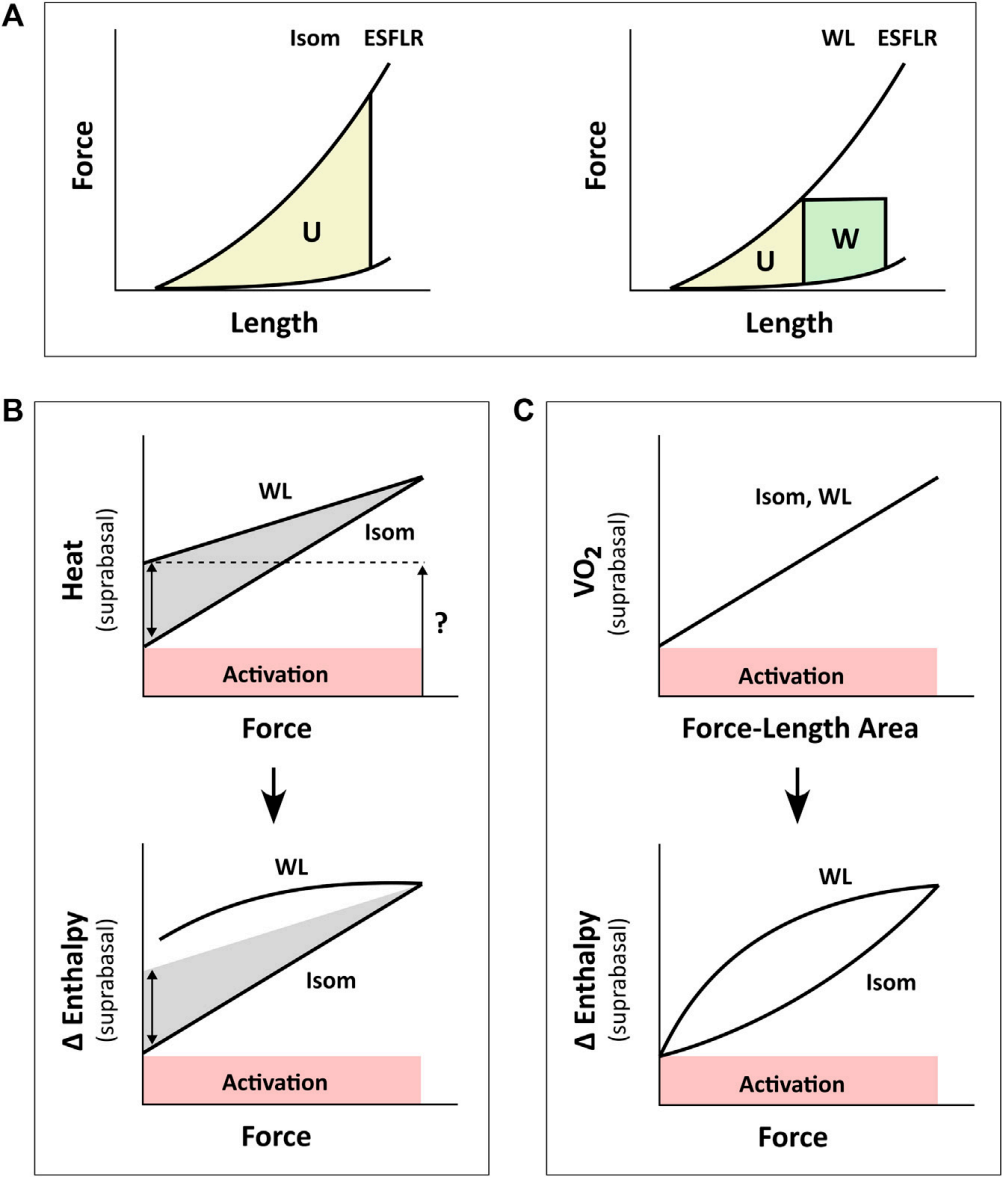
Inconsistency between studies in the estimation of activation energy from isometric contractions and work-loop contractions. Drawings in panel B are based from C. L. Gibbs (1987). Drawings in panel C are based from H. Suga (1990). **(A)** The pressure-volume area (PVA) concept formulated by H. Suga requires calculation of two areas on the pressure-volume (or force-length) plane: the unknown energy in yellow that is labelled ‘U’ and the mechanical work output in green that is labelled ‘W’. The end-systolic force-length relation (ESFLR) is formed from a series of isometric contractions (‘Isom’) or from a series of afterloaded work-loop contractions (‘WL’). We have demonstrated that the ESFLR is not the same between that obtained from isometric contractions and that obtained from work-loop contractions (Han et al., 2019a). **(B)** We have confirmed that the *y*-intercept of the isometric suprabasal heat-force relation measures cardiac activation heat (coloured red and labelled ‘Activation’) (Pham et al., 2017). We have also resolved an uncertainty of C. L. Gibbs that the *y*-intercept of the suprabasal heat-force relation obtained from WL contractions (as indicated by the question mark) is not strictly a measure of activation heat, but also contains shortening heat as signified by the double-headed arrow (Tran et al., 2017). Heat is translated to change of enthalpy (Δ Enthalpy) by adding mechanical work output (W) and, hence, results in a curved relation between Δ Enthalpy and force for WL contractions. The curve is not extrapolated to the *y*-intercept (Gibbs, 1987). **(C)** H. Suga showed that oxygen consumption (VO_2_) is linearly related to PVA (or force-length area, FLA) and is independent of whether the mode of contraction is Isom or WL. Thus, shortening heat is absent when the *x*-axis is PVA (or FLA). In contrast, shortening heat is present when the *x*-axis is pressure (or force), where it is defined by the difference in heat between the two modes of contraction. Δ Enthalpy is energetically equivalent to VO_2_. Both modes of contraction yield the same *y*-intercept, thus, again, showing that activation energy is independent of the mode of contraction, which is inconsistent with the conclusion drawn in panel B.

Around the same time, in the 1980s, H. Suga and others formulated the concept of ‘pressure-volume area’ (PVA); that is the sum of external work output and a term that was coined ‘potential energy’, which we label ‘U’ to denote an unexplained term (Figure 1A). H. Suga and others found virtually no difference in the *y*-intercept when myocardial oxygen consumption (VO_2_) was plotted against PVA for the isolated heart beating isovolumically or cyclically ejecting (Khalafbeigui et al., 1979; Suga et al., 1981; Suga et al., 1986a). The same conclusion regarding the contraction mode-independence of the *y*-intercept was obtained when PVA was substituted with its tissue analogue, force-length area (FLA), in isolated papillary muscles (Hisano and Cooper, 1987). By subtracting the basal metabolism, the remaining (suprabasal) magnitude of the *y*-intercept of the VO_2_-PVA relation was defined by H. Suga as the “*VO*_*2*_ *… used for* … *electrical activation,* and *excitation-contraction coupling involving calcium release and uptake*” (Suga et al., 1983), “*VO*_*2*_ *associated with myocardial activation, or excitation-contraction coupling*” (Suga et al., 1986a), or, in a much-abbreviated form, “*VO*_*2*_ *for EC coupling*” (Suga et al., 1988). Thus, the *y*-intercept of the suprabasal VO_2_-PVA relation measures cardiac activation metabolism, which C. L. Gibbs (1987) labelled “*activation oxygen consumption*”.

A consensus was reached, in the 1980s, that the *y*-intercept of the suprabasal heat-stress relation and that of the suprabasal VO_2_-PVA (or VO_2_-FLA) relation were equally a measure of cardiac activation energy. However, an uncertainty remains regarding the contraction mode-dependency of activation energy, i.e., the suprabasal heat-stress relation gives a higher activation heat under isotonic contractions than under isometric contractions (Figure 1B), whereas the suprabasal VO_2_-PVA relation gives virtually the same estimate of activation energy which is independent of the mode of contraction (Figure 1C). This uncertainty has not been resolved despite conversions of the *y*-axis by C. L. Gibbs from heat to change of enthalpy (Δ Enthalpy; the sum of the heat and work output, which is energetically equivalent to oxygen consumption) (Gibbs et al., 1967; Gibbs et al., 1992). C. L. Gibbs and others have even directly measured oxygen consumption (Gibbs et al., 1990) and stress-length area, SLA (Gibbs et al., 1992) in isolated papillary muscles. Likewise, H. Suga substituted PVA or FLA for the *x*-axis with systolic pressure (Nozawa et al., 1989) or force (Taylor et al., 1993) and demonstrated robust contraction mode-independence of the activation energy (Suga, 1990), as illustrated in Figure 1C. The uncertainty had involved discussion regarding the Fenn effect (Gibbs, 1987) and reconciliation with the Fenn effect when the *x*-axis is systolic pressure or force (Figures 1B,C). Fenn effect is that heat output is greater under shortening contractions than under isometric contractions (Fenn, 1924; Gibbs, 1987), as illustrated in Figure 1B. Nevertheless, whereas C. L. Gibbs and others inferred from their data that there is unlikely a Fenn effect in cardiac muscle (Holroyd and Gibbs, 1992), H. Suga and others concluded to the contrary (Nozawa et al., 1989; Taylor et al., 1993). The conclusion of H. Suga and others was supported in 1996, where the Fenn effect was observed in chemically-permeabilised cardiac trabeculae as myofibrillar ATP turnover was shown to be greater during repetitive length changes than during isometric contraction (Ebus and Stienen, 1996).

Despite this unresolved inconsistency, the use of the VO_2_-PVA concept has, since the 1990s, dominated the field of cardiac mechanoenergetics. Specifically, the activation energy estimated from the VO_2_-PVA relation has since been used to study the effects of intravenous Ca^2+^ infusion (Ohgoshi et al., 1992; Matsushita et al., 1995; Hata et al., 1998) or perfusion (Wannenburg et al., 1992), Ca^2+^ overload (Tsuji et al., 2001), positive (Wu et al., 1989; Takeuchi et al., 2001) or negative (Nakayama et al., 1997) inotropic interventions that target Ca^2+^-specific receptors, ryanodine-treated hearts having Ca^2+^-leaky sarcoplasmic reticula (Takasago et al., 1993), and inhibition of the sarcoplasmic reticular Ca^2+^-ATPase (Misawa et al., 2001). It has also been used in studies of cardiac diseases that inferred impaired energy utilisation for intracellular Ca^2+^ handling in hyperthyroid hearts (Goto et al., 1990), hypertensive-hypertrophied hearts (Kameyama et al., 1998; Morii et al., 1998), acute endotoxemia (Aghajani et al., 2004), diabetes (Abe et al., 2002; Ramanathan et al., 2002; How et al., 2006), following knockout of the sarcoplasmic reticulum (Zhang et al., 2012; Mitsuyama et al., 2013; Boardman et al., 2014), patients with ischemic heart disease (Takaoka et al., 1993), as well as in the exploration of cellular Ca^2+^-handling targeted therapeutic strategies (Sakata et al., 2006; Hafstad et al., 2007; Hafstad et al., 2013). The use of the VO_2_-PVA relation in the assessment of Ca^2+^ handling in excitation-contraction coupling remains unquestioned in recent experimental studies (Obata et al., 2018; Obata et al., 2019; Obata et al., 2020), review articles (Bastos et al., 2019; Hieda and Goto, 2020), and a book chapter (Westerhof et al., 2019).

Unlike the VO_2_-PVA relation which has been taken to be contraction mode-independent, the use of the heat-force relation in estimating activation heat (*y*-intercept) is limited to only isometric contractions (Figure 1B). Specifically, the activation heat estimated from the isometric heat-force relation has been used to study the effects of temperature (Loiselle, 1979; Johnston et al., 2016), stimulus frequency (Gibbs and Gibson, 1970a; Han et al., 2010; Johnston et al., 2016), aging (Kiriazis and Gibbs, 2001), extracellular Ca^2+^ concentration (Han et al., 2010), high-salt diet (Tran et al., 2016) and fish oil supplementation (Goo et al., 2014). It has also been used in studies of cardiac diseases including anthracycline-induced cardiomyopathy (Gibbs et al., 1984; Gibbs et al., 1986), overloaded-induced ventricular hypertrophy (Gibbs et al., 1992; Kiriazis et al., 1992), and systemic- (Han et al., 2014a) and pulmonary- (Han et al., 2018; Pham et al., 2018) hypertension-induced hypertrophy, and diabetes (Han et al., 2014b).

We have recently confirmed, using a pharmacological agent, blebbistatin, which specifically inhibits crossbridge cycling, that the *y*-intercept of the heat-force relation obtained non-pharmacologically from cardiac muscles contracting isometrically quantifies activation heat (Pham et al., 2017). In contrast, from muscles presented with contractions that involve shortening including isotonic or work-loop contractions, the higher *y*-intercept that results from the work-loop heat-force relation contains the heat consequent to muscle shortening, termed the ‘heat of shortening’ in cardiac muscle (Tran et al., 2017), in line with the Fenn effect, as illustrated by the double-headed arrow in Figure 1B. We have also presented the concept of ‘cardiac end-systolic zone’ that confirms the contraction mode-dependence of the cardiac end-systolic force-length relation (Han et al., 2019a). This concept applies also on the heat-force plane (Tran et al., 2020). The underlying concept is that the end-systolic zone bounds the possible end-systolic force-length points that result from combinations of preload (or initial/diastolic muscle length) and afterload. That is, every end-systolic force-length relation, established from a set of end-systolic points, is not the same between that arising from isometric contractions and that arising from isotonic or afterloaded work-loop contractions (Figure 1A). The contraction mode-dependency of the cardiac force-length relation is more apparent at long muscle lengths (high preloads).

The present study is motivated by two considerations. The first is the applicability of the VO_2_-PVA concept for assessment of activation energy (described above) given widespread adoption of this concept in the literature and clinic. The second is our recent understanding of the cardiac mechanoenergetics framework gained from our previous studies regarding the so-called ‘potential energy’ by H. Suga (Han et al., 2012a; Han et al., 2012b; Loiselle et al., 2021), cardiac shortening heat (Tran et al., 2017) and contraction-mode dependency of the force-length relation that constitutes the ‘end-systolic zone’ framework (Han et al., 2019a; Han et al., 2019b; Han et al., 2021). That is, in our previous studies constructing the ‘end-systolic zone’ framework, we examined the force-length and heat-force relations. In this study, with a different dataset, we extend our examination and leverage the insights gained from the end-systolic zone framework to reconcile the contraction mode-dependency observed on the heat-force plane (Figure 1B) with the contraction mode-independency observed on the VO_2_-PVA (or, equivalently, Δ Enthalpy-FLA) plane (Figure 1C). Here, we focus on Δ Enthalpy-FLA relations, and are able to achieve resolution of the 60-year-old uncertainty regarding the contraction mode-dependency of cardiac activation energy. Based on findings in the present study, we comment on directions of future use of the activation energy assessed from H. Suga’s VO_2_-PVA concept.

## Materials and methods

### Ethical approval

Rats (Wistar, male, 9–10 weeks of age and weighed 250 g–350 g) were obtained from the Vernon Jansen Unit of The University of Auckland, where they were reared in cages (22°C) on a 12-h light-dark cycle and had *ad lib* access to standard chow and water. On the day of experimentation, a rat was anaesthetised with isoflurane (1,000 IU kg^-1^), then cervically dislocated before the heart was quickly removed and placed into chilled (4°C) Tyrode solution. This procedure was approved under ethics R2006 by the Animal Ethics Committee of The University of Auckland.

### Muscle preparation

The excised heart was perfused, via the Langendorff mode, with Tyrode solution (room temperature, 22°C) containing 20 mmol L^-1^ 2,3-butanedione monoxime. The Tyrode solution contained, in mmol L^-1^, 130 NaCl, 6 KCl, 1 MgCl_2_, 0.5 NaH_2_PO_4_, 0.3 CaCl_2_, 10 HEPES, 10 glucose. Its pH was adjusted using Tris to 7.4 and the solution was bubbled with 100% oxygen. The cannulated heart was completely submerged in the same Tyrode solution in a dissection dish. The heart was cut open at the septum to expose the interior of the left ventricle, and trabeculae were dissected. A trabecula was then mounted in our microcalorimeter (Taberner et al., 2011; Taberner et al., 2018) where it was superfused with oxygenated Tyrode solution with a higher concentration of CaCl_2_ at 1.5 mmol L^-1^. The rate of flow of Tyrode superfusate was electronically maintained at 0.6 μL s^-1^. This rate provides sufficient flow of oxygenation to the trabecula (Han et al., 2011) while maximising the thermal signal-to-noise ratio of the microcalorimeter (Johnston et al., 2015; Taberner et al., 2018).

In the microcalorimeter, two arrays of thermopiles, located exterior to the measurement chamber upstream and downstream of the mounted trabecula, measured the temperature change of the flowing superfusate. Electrical stimulation was provided by a pair of platinum electrodes, located proximate to the measurement chamber. The trabecula was stimulated at 2 Hz and was allowed to achieve equilibration, typically for at least 1 h, until a steady state of force development was reached. The trabecula was stretched to reach its optimal length (*L*_*o*_) where maximal developed force was achieved. The entire system was enclosed within an insulated hood to render the calorimeter light-proof and thermally isolated. The temperature within the enclosure was maintained at 37°C by temperature controllers located on the top and at the bottom of the calorimeter measurement unit, and by a controller located on the optical table on which the entire calorimeter system was mounted.

### Work-loop control system

The trabecula was mounted between two platinum hooks in the measurement chamber of the microcalorimeter. The upstream hook was connected to a length controller motor, and the downstream hook was connected to a force transducer. The mounted trabecula was subjected to contract isometrically or to perform work-loops using our control systems. The description of both our isomeric and work-loop control systems has been detailed elsewhere (Taberner et al., 2011; Taberner et al., 2019). Briefly, isometric contractions were achieved by using the upstream length controller motor to compensate only for tiny deflections of the downstream force transducer, thereby maintaining muscle length constant during contractions. In work-loop control mode, on elicitation of a muscle twitch, the length controller first allows isometric force development, then transitions to isotonic mode to allow the muscle to shorten at any user-selected, constant, afterload within the range between passive force and peak isometric force. When the muscle can no longer sustain the prescribed afterload without being re-stretched, the length controller transitions back to isometric mode to allow the muscle to relax isometrically. Finally, the length controller re-stretches the muscle back to its initial length.

### Experimental protocols

Experiments commenced typically 1 h following mounting of the trabecula by which time a stable thermal environment within the enclosure had been attained. The trabecula was stimulated to contract at 4 Hz. Force, length and rate of heat were recorded simultaneously. Two experimental protocols were performed, which were designed, respectively, to establish end-systolic force-length relations under work-loop contractions and under isometric contractions, together with their equivalent heat-force relations.

The first protocol was designated the “afterloaded work-loop contraction protocol” which involved a series of work-loop contractions at six different afterloads where each bout of afterloaded work-loops was interspersed with isometric contractions. Six afterloads were chosen to encompass the range from maximal at peak isometric twitch force to minimal in the vicinity of passive force. Steady states of work-loop and heat output were reached within 2 min per bout. This protocol was performed at three initial (end-diastolic) muscle lengths (*L*_*o*_, 0.95 *L*_*o*_ and 0.90 *L*_*o*_) to obtain three work-loop end-systolic force-length relations and their corresponding heat-force relations.

The second protocol was designated the “preloaded isometric contraction protocol” which required the trabecula to contract isometrically at six different lengths, ranging from *L*_*o*_ (where developed force was maximal) to *L*_min_ (around 0.75 *L*_*o*_, where developed force was minimal, i.e., near zero). Stimulation was halted between each length change step to allow measurement of the baseline of rate of heat from the muscle in its quiescent state. From both force and heat measurements at these six lengths, the isometric end-systolic force-length relation and the isometric heat-force relation were established. The *y*-intercept of the latter relation provides the measure of activation heat.

### Determination of active heat

Upon completion of each experiment, stimulation was halted, leaving the trabecula quiescent in the measurement chamber. This facilitated quantification of two artefactual sources of active heat measurement. First, the heat artefact arising from the change of basal rate of heat output as a result of change of muscle length during active work-loop contractions, which accounted for the Feng effect (Garrett et al., 2021), was quantified. This measurement was conducted using the length controller to electronically oscillate the quiescent trabecula between *L*_*o*_ and the minimal length. Second, the trabecula was then electronically re-located further downstream of the measurement chamber to be positioned distal to the thermopile arrays. In the absence of the trabecula between the two thermopile arrays, the heat artefact resulting from electrical stimulation was quantified. Measurements of supra-basal, active, muscle heat production were then corrected retrospectively by subtraction of both sources of heat artifact.

### Geometric measurement of trabeculae

The geometry of each trabecula was measured at *L*_*o*_ using a microscopic graticule from both the top view and the perpendicular view via a 45° mirror located exterior to the measurement chamber. Muscle cross-sectional area was assumed to approximate an ellipse. Of the 10 trabeculae studied, their average top diameter was 305 µm ± 64 µm (mean ± standard deviation) and the average perpendicular diameter was 303 µm ± 82 μm, giving an average cross-sectional area of 0.076 mm^2^ ± 0.036 mm^2^. Their average length was 3.25 mm ± 0.46 mm.

### Quantifications of measured variables

Mechano-energetic data were analysed using custom-written code within the Matlab interface. Muscle force was converted to stress by dividing by cross-sectional area. Muscle length was expressed relative to the optimal muscle length (i.e., *L*/*L*_*o*_). Preload was defined as the diastolic stress at the initial length. A stress-length work-loop has four distinct phases: isometric contraction, isotonic shortening, isometric relaxation and isotonic re-lengthening (Taberner et al., 2011). Afterload was defined as the stress at which the muscle transitioned from the isometric contraction phase to the isotonic shortening phase. The width of the loop represented the extent of muscle shortening. The area within the loop quantified the mechanical work performed by the muscle. Rate of heat output was converted to heat density by dividing by stimulus frequency (4 Hz) and muscle volume. Change of enthalpy equated the sum of mechanical work and suprabasal heat output; the latter was partitioned into activation heat and crossbridge heat.

### Simulation of work-loops

Using mechano-energetics data obtained from the two experimental protocols described above, we constructed a geometrical model that enabled simulation of work-loops at any combination of preload and afterload. The geometrical model was based on the cardiac ‘end-systolic zone’ on the stress-length plane (Han et al., 2019a). This zone contains the end-systolic points for any combination of preloaded and afterloaded work-loops within the range of muscle length from *L*_min_ to *L*_*o*_. Thus, for any given preload (or initial muscle length) and afterload, the resulting end-systolic point within the end-systolic zone was located. Simultaneous measurements of heat allowed the delineation of the zone on the heat-stress plane–the energetics equivalent of the end-systolic zone; that is, any end-systolic point on the stress-length plane has an equivalent unique point on the heat-stress plane (Tran et al., 2020). Together, these two frameworks form the geometric model. Using this model, for any given combination of preload and afterload, mechanical work output (the area of the stress-length work-loop) and change of enthalpy (the sum of work and heat) were calculated. Suga’s ‘potential energy’ (U) and, hence, stress-length area (SLA), which is the sum of work and ‘U’, were also calculated.

### Statistical analyses

Stress and heat were plotted as functions of relative muscle length, and fitted using polynomial regression. The regression lines were averaged, and the differences among regression lines were tested, using the Random Coefficient Model within Proc Mixed implemented within the SAS software interface. Statistical significance of a difference was declared when *p* < 0.05.

## Results

**FIGURE 2:**
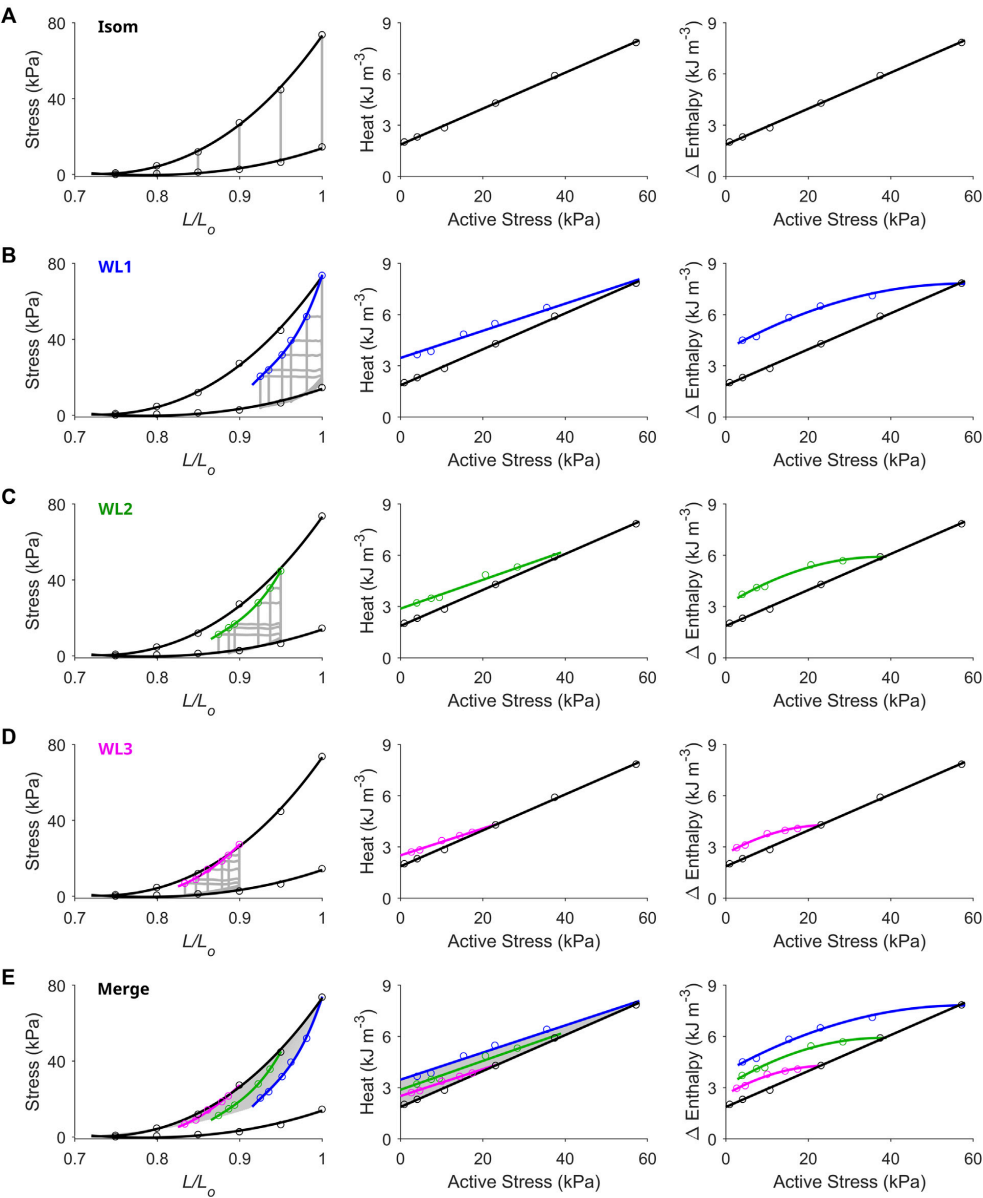
Unique end-systolic points result from unique combinations of initial length, afterload and mode of contraction. Data were obtained from a representative trabecula. **(A)** The trabecula contracted isometrically at six initial lengths. On the left-hand column, the isometric (Isom) end-systolic stress-length relation (upper thick line) and the isometric passive stress-length relation (lower thick line) are fitted to the six isometric contractions (grey lines) at end-systole (top circles) and at end-diastole (bottom circles), respectively. On the middle column, the isometric heat-stress relation is fitted to the six data points, where the *y*-intercept is the measure of activation heat. These Isom relations (black) are transcribed to the subsequent panels in the middle column. **(B)** The trabecula performed work-loop contractions (grey loops) at six afterloads (one of which was maximal at the isometric stress) and at the initial length of *L*_*o*_ (WL1). The WL end-systolic stress-length relation is fitted to the six end-systolic points (blue). The WL heat-stress relation is obtained by fitting to the six data points (blue). **(C)** Same as in panel B, but with the initial length of the trabecula set at 0.95 *L*_*o*_ (green). **(D)** Same as in panels B and C, but with the initial length of the trabecula set at 0.90 *L*_*o*_ (magenta). **(E)** All Isom and WL relations from the preceding panels are merged. The area highlighted in grey is the end-systolic zone (left-hand panel), which has its energetics equivalent zone on the heat-stress plane (middle panel). Each of the end-systolic points (circles) within the end-systolic zone maps to its energetic equivalent point within the heat-stress zone. In the middle panel, the difference between each of the WL heat-stress relations and the isometric heat-stress relation quantifies shortening heat (Fenn effect). The right-hand column of all panels plots the Δ enthalpy-stress relations. Note that Δ enthalpy is the sum of heat and work, where under isometric contractions (panel A), Δ enthalpy is entirely liberated as heat.

**FIGURE 3:**
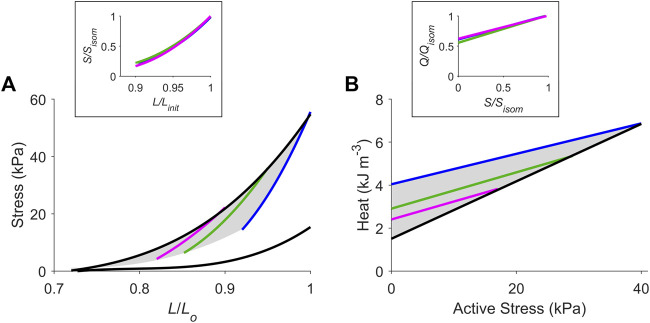
The cardiac end-systolic zone on the stress-length plane and its energetics equivalent on the heat-stress plane. Average relations from all 10 trabeculae. **(A)** Average isometric end-systolic stress-length relation (black) and average work-loop end-systolic stress-length relation at initial lengths of *L*_*o*_ (blue), 0.95 *L*_*o*_ (green) and 0.90 *L*_*o*_ (magenta). The end-systolic zone is coloured in grey. In the inset, the three work-loop end-systolic stress-length relations are each self-normalised to their respective isometric values (subscripted as “isom” and as “init” that denotes initial length). No statistical significance among the three self-normalised relations were detected. **(B)** Average isometric heat-stress relation (black), where the *y*-intercept is the measure of activation heat. Average work-loop heat-stress relations at initial lengths of *L*_*o*_ (blue), 0.95 *L*_*o*_ (green) and 0.90 *L*_*o*_ (magenta). The energetics equivalent of the end-systolic zone (heat-stress zone) is coloured in grey. In the inset, the three work-loop heat-stress relations are each self-normalised to their respective isometric values (Q denotes heat). No statistical significance among the three self-normalised relations were detected.

**FIGURE 4:**
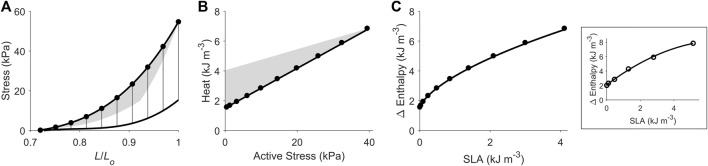
Replicating mechanoenergetics results under isometric contractions. Using the end-systolic zone framework (grey zones in panels A and B), isometric contractions at various muscle lengths are replicated. **(A)** Replicated isometric contractions (vertical lines), where the resulting end-systolic points are indicated by filled circles. The isometric end-systolic stress-length relation and the isometric passive stress-length relation are transcribed from Figure 3. **(B)** The resulting points on the heat-stress plane are indicated by filled circles. The isometric heat-stress relation is transcribed from Figure 3. **(C)** The end-systolic zone framework computes the relation between change of enthalpy (Δ Enthalpy) and stress-length area (SLA) under isometric contractions. SLA is calculated for each of the isometric contractions as illustrated in Figure 1A, where stress is taken as zero at 0.72 *L*_*o*_ (based on our data; see Figure 2). For isometric contractions, external mechanical work output is zero and, hence, SLA consists entirely of ‘U’ and Δ Enthalpy consists entirely of heat. In the inset, data (open circles) from a representative trabecula are fitted by a second-order polynomial.

**FIGURE 5:**
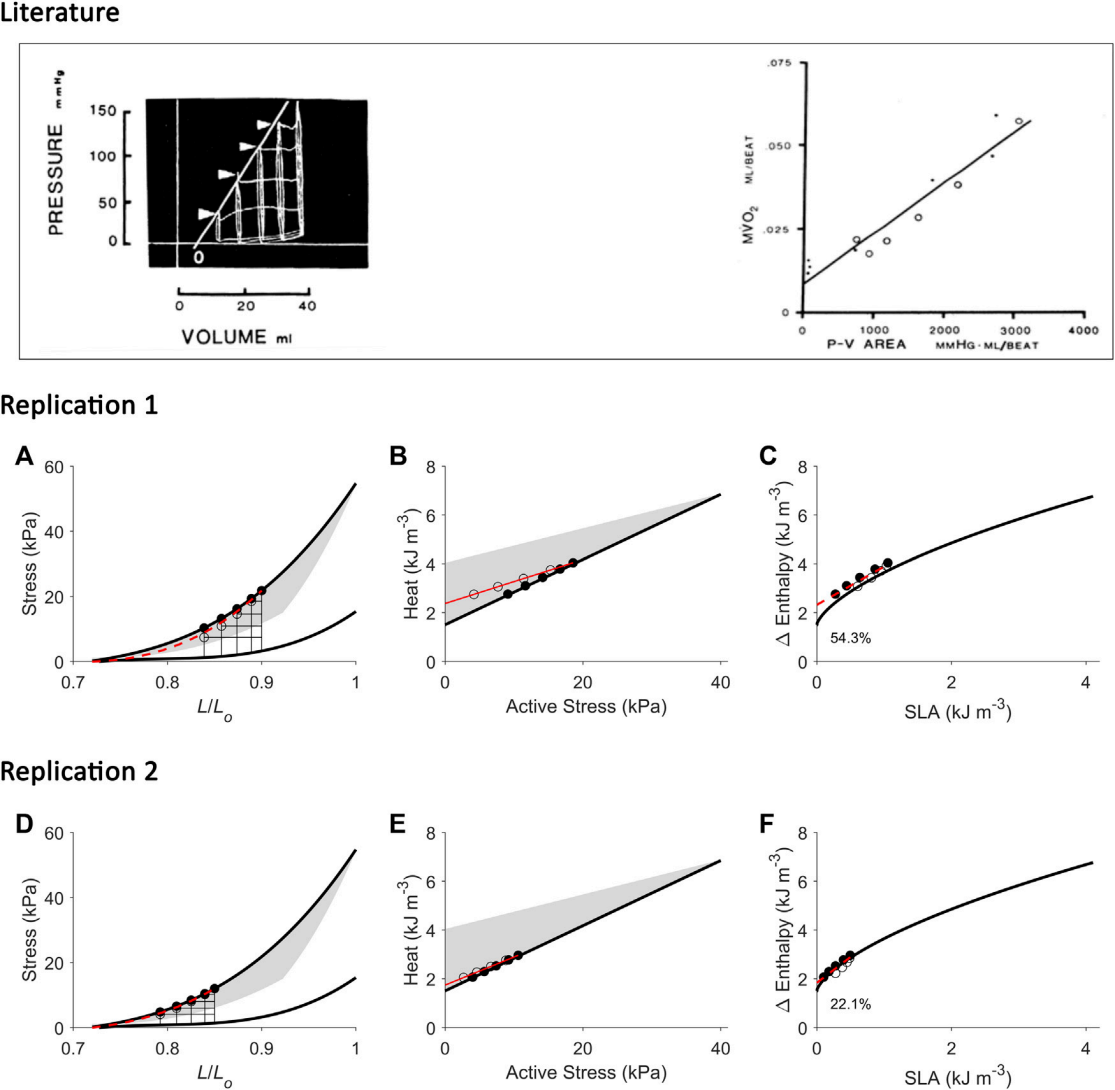
Replicating mechanoenergetics results of (H). Suga. Literature figures of H. Suga and colleagues in the first report of their VO_2_-PVA concept (Khalafbeigui et al., 1979) are reprinted with permission from The American Physiological Society via Copyright Clearance Center. Their data show pressure-volume loops superimposed between isovolumic and ejecting contractions. It appears that there are five isovolumic contractions superimposed with four ejecting contractions where their end-systolic points are indicated by the four arrowheads. The end-systolic pressure-volume relation was fitted with linear regression ‘*regardless of end-diastolic volume and mode of contraction’*. They also fitted the data using linear regression to relate myocardial oxygen consumption (MVO_2_) to pressure-volume area (PVA) (closed circles: isovolumic contractions; open circles: ejecting contractions). **(A)** Our replication of the literature results using five isometric contractions (end-systolic points: filled circles) and four work-loop contractions (end-systolic points: open circles) to obtain an end-systolic stress-length relation fitted to all nine end-systolic points regardless of the initial length and mode of contraction (dotted red line). This allows calculation of ‘U’ for the plot in panel C. **(B)** Equivalent heat-stress points of the replicated end-systolic points in panel A, where the red line is fitted only to the four work-loop heat-stress points (open circles) to illustrate contraction-mode dependency (consistent with experimental data shown in Figures 2, 3). **(C)** The resultant Δ Enthalpy-SLA points are fitted with linear regression regardless of mode of contraction (broken red lines). The resultant *y*-intercept of the linear relation is 54.3% higher than the *y*-intercept of the isometric relation (solid line; transcribed from Figure 4C). **(D–F)** Same replications as in panels A–C except that the maximal initial length is 0.85 *L*_*o*_ to illustrate that the lower the initial length (or preload), the closer the fitted end-systolic stress-length relation (broken red line; panel D) is to the isometric end-systolic stress-length relation (thick black line). Likewise, the resultant *y*-intercept of the linearly-fitted Δ Enthalpy-SLA relation (broken red line; panel F) is closer to that of the isometric case (22.1%). Small, but crucial, details are convincingly replicated. First, the end-systolic point of the work-loop at the lowest afterload is lower than that of the isometric contraction of equivalent end-systolic length (panels A and D). Second, the work-loop at the lowest afterload encompasses the end-systolic lengths of the work-loops at the three higher afterloads (panels A and D). Lastly, on the Δ Enthalpy-SLA plane, the work-loop points sit lower than the isometric points (panels C and F).

**FIGURE 6:**
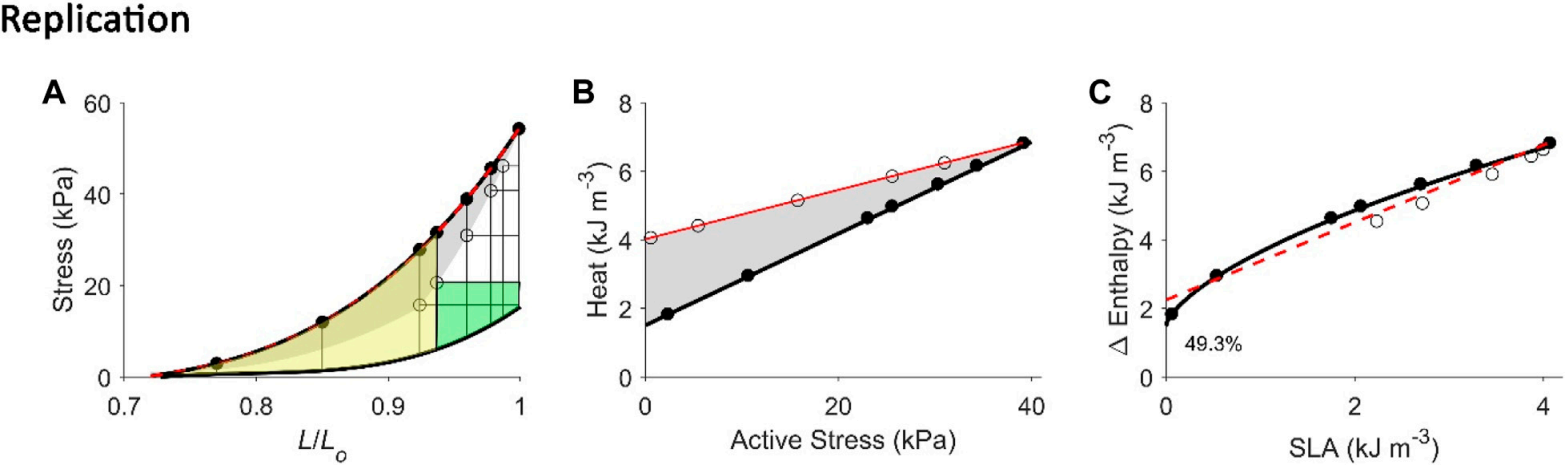
Replicating mechanoenergetics results where work-loop contractions are performed at optimal length. Our replication of Figure 4 of Hisano and Cooper (1987). Their experiments were conducted on an isolated papillary muscle undergoing isometric contractions at various lengths and afterloaded work-loop contractions at the optimal length. Four of seven isometric contractions coincide with the end-systolic length of the four lower work loops. A selected work loop is coloured green to indicate musclework output; the adjacent area that was taken by the authors as “U” is coloured yellow(same colour convention as in Figure 1A). Note that for this selected work loop, its end-systolic point is lower than that achieved by the isometric contraction at the matched end-systolic length. The authors fitted a linear line to illustrate contraction mode independence of the relation betweenmyocardial oxygen consumption and peak force, as well as with force-length area. **(A)** Our replication of their results, consisting of five afterloaded work loops at the initial length of Lo (end-systolic points indicated by open circles) and seven isometric contractions (end-systolic points indicated by filled circles) where four of them coincide with the end-systolic length of the four lower work loops. The selected work loop is coloured to illustrate areas representing work (green) and “U” (yellow). The end-systolic line (broken red line) is fitted only to the isometric contractions, as is consistent with their approach. **(B)** Equivalent heatstress points of the replicated end-systolic points in (A). The red line is fitted to the work-loop heat-stress points (open circles) that sit higher than the isometric heat-stress points (filled circles) for a given stress, which resembles their data above. This case is also obtained in our experiments, as graphed in Figure 2B. **(C)** The resultant Δ Enthalpy-SLA points are fitted with linear regression (broken red line) regardless of the mode of contraction. The resultant y-intercept of the linear relation is 49.3% higher than the y-intercept of the isometric relation (solid line; transcribed from Figure 4C). Two reassuring details of our replication are that the work loop at the lowest afterload encompasses the end-systolic lengths of the work loops at the four higher afterloads (panel A), and the work-loop points sit higher than the isometric points on the heat-stress plane (panel B).

**FIGURE 7:**
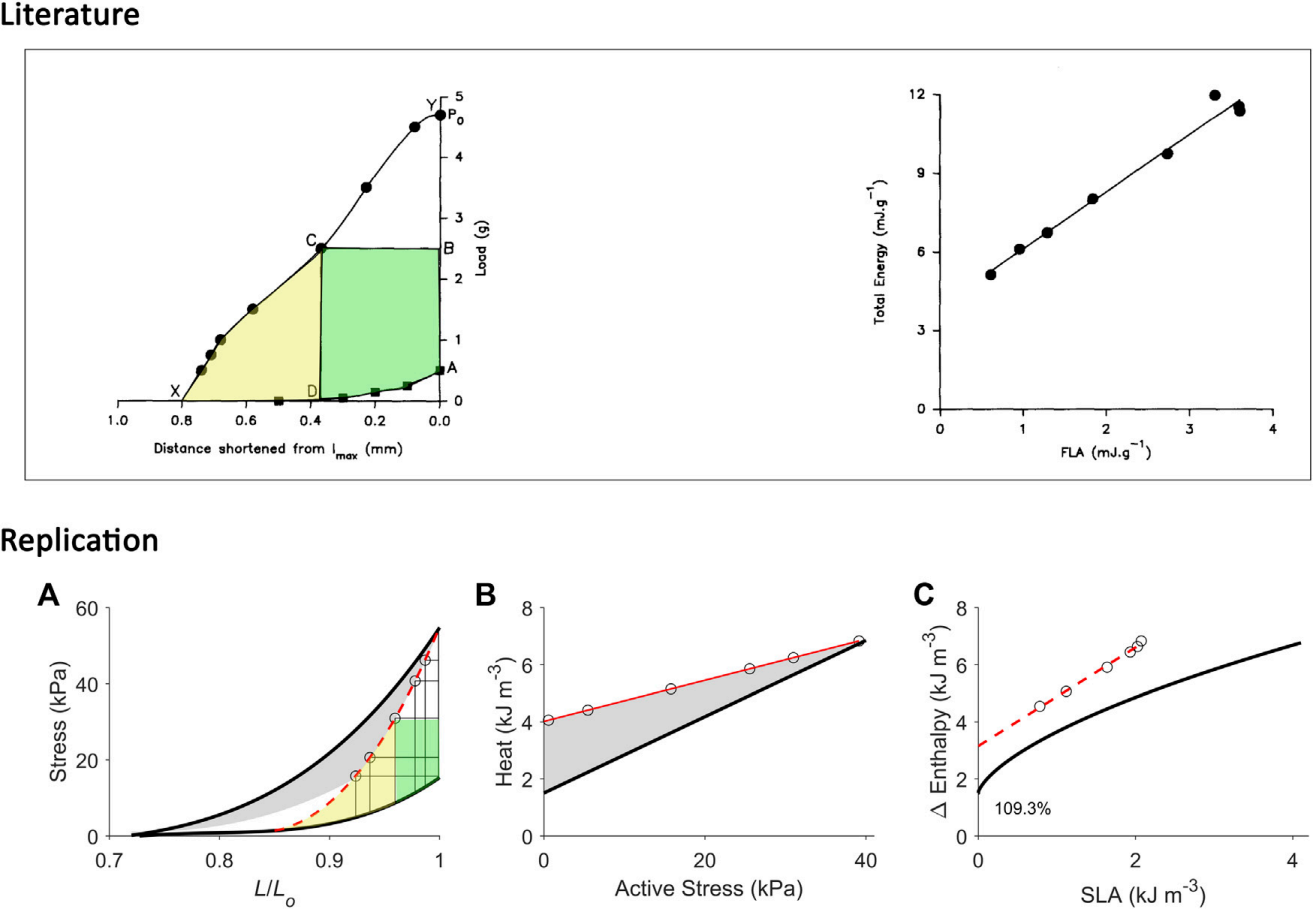
Replicating mechanoenergetics results for afterloaded work-loop contractions at optimal length. Literature figures show data of C. L. Gibbs and colleagues; reproduced from Kiriazis et al. (1992) with permission from Springer Nature via Copyright Clearance Center. Their experiments were conducted on an isolated papillary muscle shortening at various loads that was set at an initial length that was optimal (‘*l*_max_’). *P*_*o*_ is the isometric force at *l*_max_. The end-systolic points of afterloaded isotonic (work-loop) contractions are indicated by filled circles, and were connected to obtain the curved work-loop end-systolic force-length relation (the line connecting Y-C-X). For the selected loop, the work is coloured green and the adjacent area that denotes ‘U’ is coloured yellow. They fitted a linear line to data to describe the relation between ‘total energy’ (enthalpy) and FLA (force-length area). **(A)** Our replication of their results, consisting of five afterloaded work-loops at the initial length of *L*_*o*_ (end-systolic points indicated by open circles) and an isometric contraction at *L*_*o*_ (to replicate ‘*P*_*o*_’). The work-loop end-systolic stress-length relation (broken red line) is obtained by fitting to all end-systolic points (to replicate the line connecting Y-C-X). The fitted red line intersects the passive stress-length relation at 0.85 *L*_*o*_ and this length is taken as the minimal length where stress is zero, to allow calculation of ‘U’ (the area coloured yellow). **(B)** Equivalent heat-stress points of the replicated work-loop end-systolic points in panel A (open circles). **(C)** The resultant Δ Enthalpy-SLA points (open circles) for the work-loop contractions are fitted with linear regression (broken red line), with the resultant *y*-intercept 109.3% higher than that of the isometric relation. In all panels A–C, the isometric relations (thick black lines) are transcribed from Figure 4.

**FIGURE 8:**
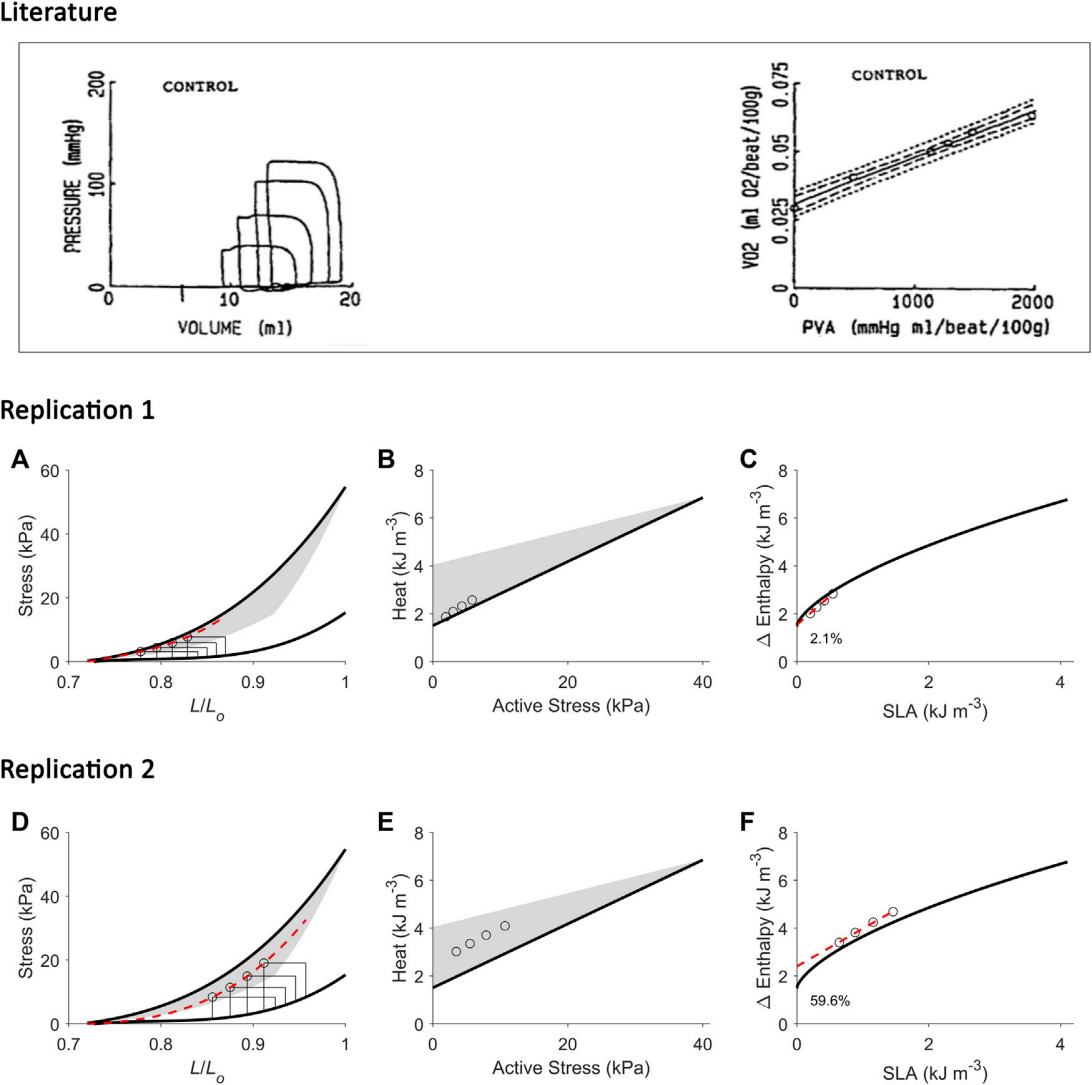
Replicating mechanoenergetics results for work-loops with simultaneous increase of preload and afterload. Literature figures show data of H. Suga and colleagues; reproduced from Wu et al. (1989) with permission from Springer Nature via Copyright Clearance Center. In their experiments, an excised cross-circulated heart was subjected to ‘*variously loaded ejecting contractions*’. The resultant pressure-volume loops had initial volume (or preload) greater at greater afterload. Linear correlation between left-ventricular oxygen consumption (VO_2_) and pressure-volume area (PVA) was obtained. **(A)** Our replication of their results, consisting of four preloaded-afterloaded work-loops (end-systolic points indicated by open circles) with the largest work-loop set at the initial length of 0.87 *L*_*o*_. The end-systolic stress-length relation (broken red line) is obtained by fitting to the four work-loop end-systolic points, with the minimum length set at 0.72 *L*_*o*_. **(B)** Equivalent heat-stress points (open circles) of the replicated work-loop end-systolic points in panel A. **(C)** The resultant Δ Enthalpy-SLA points from the four work-loops (open circles) are fitted with linear regression (broken red line). The resultant *y*-intercept is 2.1% higher than that of the isometric relation. (D-F) Same replications as in panels A–C but at greater initial lengths to illustrate that the greater the initial length (or preload), the further the fitted end-systolic stress-length relation (broken red line; panel D) falls from the isometric end-systolic stress-length relation (thick black line). Likewise, the resultant *y*-intercept of the linearly-fitted Δ Enthalpy-SLA relation (broken red line; panel F) is further from the isometric case (59.6%). In all panels A–F, the isometric relations (thick black lines) are transcribed from Figure 4. Our replication includes a detail on the end-systolic lengths of work-loops at the higher afterloads that intersect the shortening trajectory of the work-loops at the lower afterloads (panels A and D).

**FIGURE 9:**
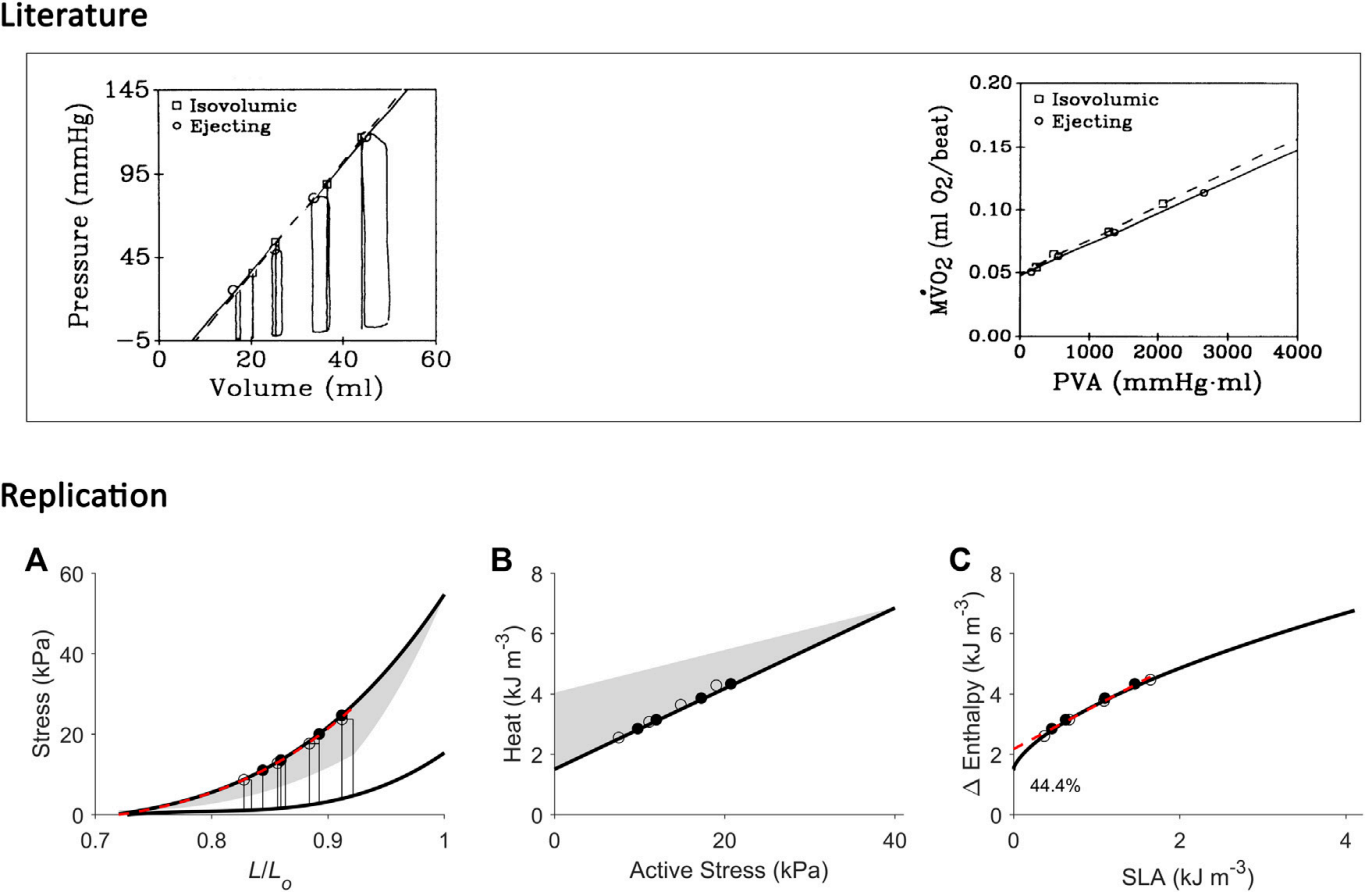
Replicating mechanoenergetics results for work-loop contractions at high afterloads. Literature figures are reproduced from Burkhoff et al. (1991) with permission from The American Physiological Society via Copyright Clearance Center. In their experiments, an isolated heart was subjected to four isovolumic contractions and four ejecting (work-loop) contractions at high afterloads, the end-systolic points of which are indicated by squares and circles, respectively. End-systolic pressure-volume relations were obtained by fitting to the respective end-systolic points. Likewise, the data of myocardial oxygen consumption (MVO_2_) and pressure-volume area (PVA) were fitted, respectively. “*Neither ESPVR nor MVO*_*2*_ *-PVA relation differed significantly between isovolumic and ejecting conditions*”. **(A)** Our replication of their results, consisting of four high-afterloaded work-loops (end-systolic points indicated by open circles) and four isometric contractions (end-systolic points indicated by filled circles). The end-systolic stress-length relation (broken red line) is obtained by fitting to all eight end-systolic points, regardless of mode of contraction, with the minimum length set at 0.72 *L*_*o*_. **(B)** Equivalent heat-stress points of the replicated eight end-systolic points in panel A. **(C)** All the resultant eight Δ Enthalpy-SLA points are fitted with linear regression (broken red line). The resultant *y*-intercept is 44.4% higher than that of the isometric relation. In all panels A–C, the isometric relations (thick black lines) are transcribed from Figure 4. Comparable details of our replications of the literature data are demonstrated by the order of appearance of the isometric and work-loop end-systolic points (panel A) and on the Δ Enthalpy-SLA plane (panel C).

The results are separated into two sections. The first presents our experimental results from which we established our ‘cardiac end-systolic zone’ framework consisting of a family of stress-length relations and an equivalent family of suprabasal (active) heat-stress relations; both demonstrate contraction mode-dependency (Figures 2, 3). In the second (Figures 4–9), we used the framework to replicate mechanoenergetics results gleaned from the literature in order to understand literature findings that purport to show contraction mode-independency and that attempted to calculate the ‘potential energy’ term (‘U’). For simplicity, given that we consider only suprabasal energy and not basal energy, both ‘suprabasal heat’ and ‘suprabasal change of enthalpy’ (the sum of work and suprabasal heat) are hereinafter simplified to ‘heat’ and ‘change of enthalpy’ (i.e., implicitly denoting suprabasal).

### Contraction mode-dependence delineates the cardiac end-systolic zone

Figure 2 presents representative results from a single trabecula for the comparison between the end-systolic stress-length relation (ESSLR) arising from isometric contractions and those associated with afterloaded work-loop contractions. The work-loop ESSLRs (WL1, WL2 and WL3; corresponding to initial lengths of *L*_*o*_, 0.95 *L*_*o*_ and 0.90 *L*_*o*_) were all located below the isometric ESSLR. The lower the initial length, the closer the work-loop ESSLR was to the isometric ESSLR. Contraction mode-dependency yields the ‘cardiac end-systolic zone’ on the stress-length plane (Figure 2E, shaded area). The end-systolic zone is bounded by three distinct stress-length relations: the upper boundary by the isometric ESSLR, the right-hand boundary by the work-loop ESSLR at *L*_*o*_, and the lower boundary by a series of work-loop contractions where each afterload coincides with the prevailing level of passive stress at any given preloads (i.e., isotonic shortening at zero active stress). The zone contains unique end-systolic points resulting from unique combinations of initial length (or preload), afterload and mode of contraction. A zone equivalent to the end-systolic zone was obtained on the heat-stress plane, which contains the three work-loop heat-stress relations, where the work-loop heat-stress relation at *L*_*o*_ serves as the upper boundary and the isometric heat-stress relation serves as the lower boundary. Each end-systolic point within the end-systolic zone on the stress-length plane maps to its energetic equivalent point within the heat-stress zone. The Δ enthalpy-stress relations are shown in the right column of Figure 2 illustrating the greater energy consumption associated with shortening contractions relative to isometric contractions (Fenn effect). The difference in heat between each of the work-loop relations and the isometric relation quantifies shortening heat.

Average relations from all 10 trabeculae are shown in Figure 3, where the end-systolic zone and its energetic equivalent are coloured grey. In the insets, the three average self-normalised relations on the stress-length plane, and their equivalents on the heat-stress plane, were not significantly different from one another. This result demonstrates that the end-systolic stress-length relation and its accompanying heat-stress relation are both independent of preload. That is, for any given initial muscle length ≤ *L*_*o*_, the shapes of these functions are the same when double-normalised. This allows us to make interpolations of end-systolic points and heat-stress points at any preload and afterload combination within the stress-length and heat-stress planes, respectively. Activation heat, as estimated from the *y*-intercept of the isometric heat-stress relation, averaged 1.49 kJ m^-3^ ± 0.26 kJ m^-3^ (Figure 3B).

### Replication of mechanoenergetics results under isometric contractions

Using our end-systolic zone framework, and by prescribing isometric contractions at various initial lengths, change of enthalpy was curvilinearly related to stress-length area (SLA), as illustrated in Figure 4. The *y*-intercept was taken as a measure of activation heat that is equivalent to the *y*-intercept of the heat-stress relation. These isometric relations are transcribed onto the subsequent figures.

### Replication of mechanoenergetics results under work-loop contractions

Using our end-systolic zone framework, we prescribed work-loop contractions under varying afterloads but at the same initial length (or preload). The initial length was set low (Figure 5) or high at *L*_*o*_ (Figures 6, 7). As in Figure 3, the lower the initial length, the closer the work-loop ESSLR was to the isometric ESSLR and, equivalently, the closer the work-loop heat-stress relation was to the isometric heat-stress relation. This condition means that the relation between change of enthalpy and SLA under work-loop contractions would be closer to that of the isometric contraction. A parallel consequence would be that the *y*-intercept of the work-loop enthalpy-SLA relation would be closer to the activation heat signified by the *y*-intercept of the isometric enthalpy-SLA relation. This is demonstrated in Figure 5, comparing between Replications 1 and 2.

Conversely, when initial length is maximum, i.e., exclusively at *L*_*o*_, the effect of contraction mode-dependence becomes evident and presents challenges in quantifying ‘U’. In this case, the end-systolic point of the work-loop contraction and that of the matched isometric contraction are no longer at the same coordinate. Thus, ‘U’ has been quantified in the literature where the upper boundary is the isometric ESSLR (Figure 6A) or is the work-loop ESSLR (Figure 7A). By comparison, the ‘U’ estimated in the latter case is smaller than that estimated from the former case. In consequence, the *y*-intercept of the enthalpy-SLA relation of the latter case is higher than that of the former. Clearly, not both can provide the same estimate of activation heat from the isometric relation (Figures 6C, 7C).

In Figure 8, in our replication of the conditions where the increase in afterload is followed by a concurrent increase in initial length (preload), we observed the difference in the estimate of *y*-intercept of the work-loop enthalpy-SLA relation to that obtained from the isometric relation. The difference is more pronounced at greater initial lengths where contraction mode-dependency is more evident (Replication 2 *versus* Replication 1).

In Figure 9, we replicated conditions of high afterloads (i.e., relative to the isometric stress at the given initial length) to demonstrate that contraction mode-dependency can appear otherwise, i.e., contraction mode-independency would be obtained at low afterloads. In this case, the work-loop ESSLR and the isometric ESSRL would appear very close, and their difference would be insignificant on the stress-length plane, on the heat-stress plane, and on the enthalpy-SLA plane. The *y*-intercept of the enthalpy-SLA relation was in the vicinity of the activation heat estimated from the isometric relation.

## Discussion

The present study examines the reconcilability of two existing approaches of estimating cardiac activation energy (or heat). One of the two approaches plots the relation between suprabasal heat and force, while the other plots the relation between VO_2_ and pressure-volume area, PVA (or, equivalently, stress-length area, SLA). The y-intercepts of these relations have been used to estimate activation heat. For simplicity, as in the Results, ‘suprabasal heat’ and ‘suprabasal change of enthalpy’ (the sum of work and suprabasal heat) are hereinafter simplified to ‘heat’ and ‘change of enthalpy’ (i.e., implicitly denoting suprabasal).

While both approaches produce estimates of activation heat/energy that align under an isometric contraction protocol, they diverge when the mode of contraction involves ejection or shortening. The principal contributor to their divergence is that the plot of the VO_2_-PVA (or, equivalently, enthalpy-SLA) relation is robust against the mode of contraction. Thus, H. Suga demonstrated that isovolumic (isometric) and work-loop contractions on the enthalpy-SLA plane both yield the same *y*-intercept and, hence, the same estimate of activation energy (Figure 1C). In contrast, C. L. Gibbs showed that the heat-force relation from work-loop contractions is positioned higher than that of isometric contractions (Figure 1B). The present study has arrived at a point of reconciliation of these two approaches following our recent affirmation that cardiac muscle liberates shortening heat (Tran et al., 2017; Tran et al., 2020) and that the cardiac force-length relation is contraction mode dependent (Han et al., 2019a). Both of these properties of cardiac muscle constitute our cardiac end-systolic zone framework (Figures 2, 3), which allows us to replicate a range of mechanoenergetics results gleaned from the literature. In these replications, the predicted *y*-intercept of the enthalpy-SLA relation was expressed as a percentage difference to that of the isometric contraction. Note that for isometric contractions, these two approaches produce the same *y*-intercept (Figure 4). From these replications, we have identified a condition where the predicted *y*-intercept of the enthalpy-SLA relation can diverge from the isometric case. This condition arises when initial muscle length (or preload) is high, which amplifies the divergence of the end-systolic force-length relation from the isometric case (i.e., contraction mode dependence). Under this condition, a decision must be made as to how to quantify ‘U’, whether the end-systolic force-length relation is fitted to all of the isometric and work-loop end-systolic points (Figure 5), to only the isometric end-systolic points (Figure 6), or to only the work-loop end-systolic points (Figure 7). These three cases encompass work-loops of varying afterloads but all having the same initial length. In the case where afterload and initial length are both varying, the same condition is again obtained for the divergence of the *y*-intercept which occurs at a greater initial length where contraction mode-dependency is more evident (Figure 8). The afterloads would have to be high relative to the isometric force at the prevailing initial lengths to obtain an end-systolic force-length relation that sits close to the isometric force-length relation (i.e., appearing contraction mode independent), in order to achieve an estimate of activation heat closer to that from isometric contractions (Figure 9).

In addition to addressing the contraction mode-dependency of activation energy, our findings also address the conversion between the VO_2_-FLA relation and the change of enthalpy-force relation. Note that the *y*-axis for both relations, change of enthalpy and VO_2_ is energetically equivalent. Considering the *x*-axis, while both FLA and force can be calculated from the force-length plot, the calculation of FLA is not always possible. For isometric contractions, calculation of FLA is always possible because FLA is equivalent to ‘U’, and can be calculated from the isometric end-systolic force-length relation over the entire working range of the muscle from minimum to the optimal length (Figure 4). For work-loop contractions, FLA can be calculated only under conditions of low preloads (Figure 5) or at high afterloads approaching the isometric contraction (Figure 9). This is due to the uncertainty of calculating ‘U’ when the isometric end-systolic force-length relation differs from the work-loop end-systolic force-length relations. Therefore, if a VO_2_-FLA relation exists, the change of enthalpy-force relation will also exist. But the reverse is not always true. Figure 11 summarises the essence of this paragraph.

The holistic assessment of our collective results, presented in the two preceding paragraphs, raises three discussion points, which we discuss below under the four specific sub-headings.

### Activation energy *versus* activation heat

We have used these terms interchangeably (comparing Figures 1B,C). Over the cycle of a twitch, the energy that is used to maintain electrochemical gradients is expended as heat when ion channels open to facilitate the flow of ions. Hence, the activation heat that is measured in our work-loop calorimeter is proportional to the input energy (VO_2_) that is used to drive the excitation processes. In this sense, activation heat and activation energy are equivalent. Our measurement of activation heat, averaged 1.49 kJ m^-3^ ± 0.26 kJ m^-3^ (Figure 3B), is consistent with the activation heat measured in our previous study using a pharmacological agent, blebbistatin, which specifically inhibits crossbridge cycling (Pham et al., 2017).

### Initial volume/length in experiments

**FIGURE 10:**
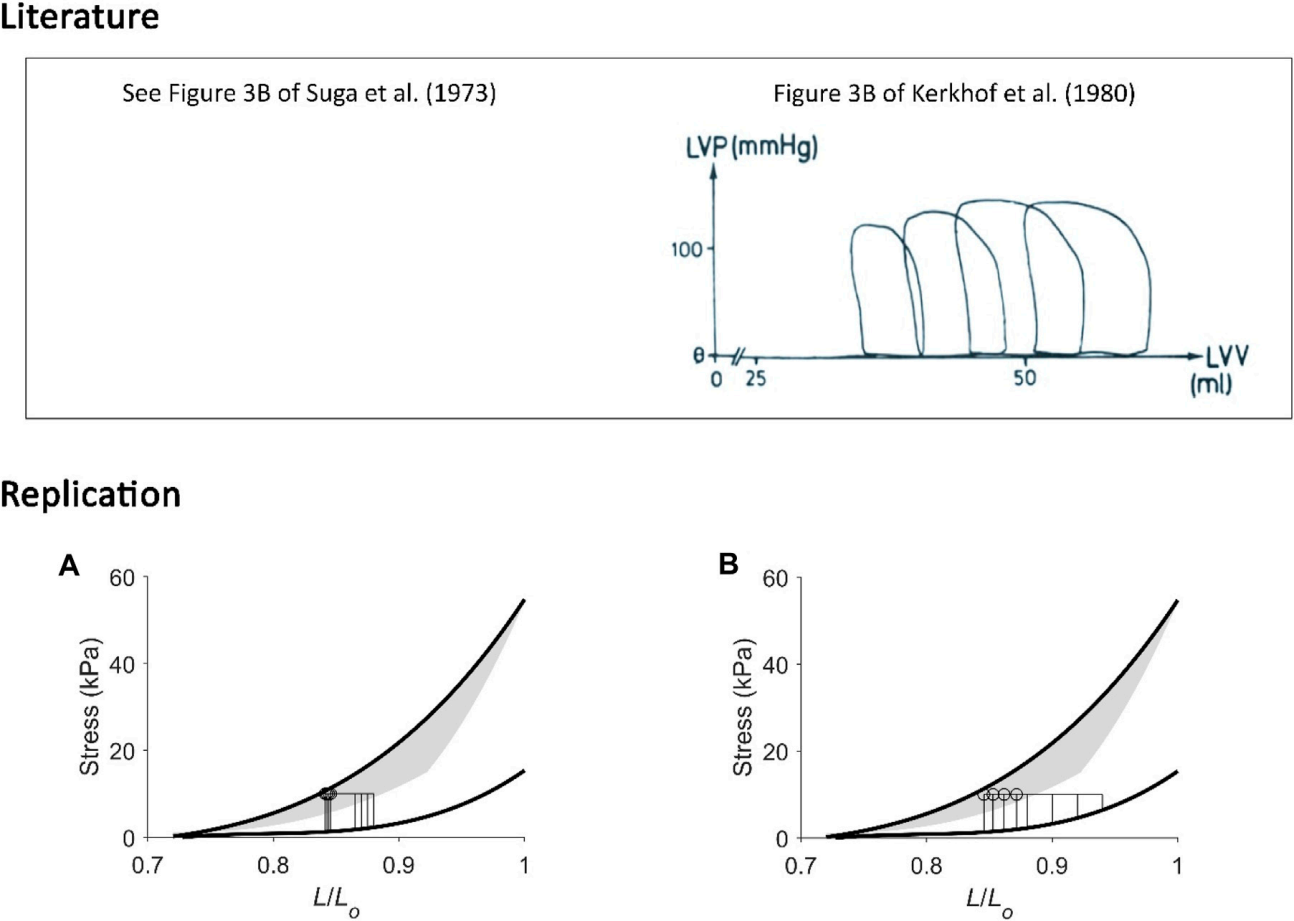
Replicating pressure-volume loops at low and at high initial volumes. **(A)** Our replication of Figure 3B of Suga et al. (1973). **(B)** Our replication of Figure 3B of Kerkhof et al. (1980), the figure of which is reproduced above in the box labelled “Literature” with permission from Springer Nature via Copyright Clearance Center. In these two unrelated experiments, isolated canine hearts were subjected to four pressure-volume loops. The mean arterial (aortic) pressure was kept constant while initial volume was varied within a lower range (left-hand panel) or a wider range at higher initial volumes (right-hand panel). In the former case **(A)**, end-systolic points from the four work loops were close to one another; they aligned close to the isovolumic pressure-volume relation, and they all share a similar “U”. In the latter case **(B)**, end-systolic points are noticeably different to one another, and our replication shows that they diverge from the isometric stress-length relation, where the greater the initial length, the further away the end-systolic point. The calculation of “U” in the latter case is ambiguous, i.e., could be by either using four different work-loop end-systolic stress-length relations or using the isometric stress-length relation with the prevailing initial length at each of the four end-systolic lengths of each work loop (as inspired from Figure 6).

First, our replications suggest that H. Suga has formulated his PVA concept based on experiments conducted at low initial volumes of the isolated heart. We address this assertion from two viewpoints, i.e., from the *x*-axis and the *y*-axis on the pressure-volume plane. Since the formulation of the PVA concept in 1979 (Khalafbeigui et al., 1979), H. Suga and others assessed the isolated canine heart typically with initial left-ventricular volumes between 15 mL (Figure 8) and 40 mL (Figure 5), although one of his earlier experiments stretched the initial volume to 60 mL in a canine heart of similar body weight (Suga and Sagawa, 1974). Initial volumes larger than 60 mL would result in work-loops that yield end-systolic points diverging from the isovolumic pressure-volume relation, confirming contraction mode-dependency and, hence, presenting an inconsistency in the calculation ‘U’ which relies on a single contraction-mode independent end-systolic pressure-volume relation (Figure 10). Under such high-volume conditions, the pressure-volume relation is no longer “load independent” as that concluded by H. Suga at low initial volumes.

In most results of H. Suga and others, the highest pressure depicted is typically from 100 mmHg (Figure 8) to 150 mmHg (Figure 5). The isovolumic pressure of the left ventricle can reach some 300 mmHg *in situ* in dogs of similar body weight, as demonstrated by an earlier study (Ullrich et al., 1954) of which H. Suga was aware (Suga et al., 1986b). Thus, H. Suga has typically studied isolated hearts under conditions of low initial volume, where the end-systolic pressure is half the peak isovolumic pressure. In our data, the peak isometric stress is halved when initial length is below 0.9 *L*_*o*_ (Figure 5). H. Suga has explicitly stated that they “*have not studied the peak isovolumic PV curve above 200–225 mmHg in the adult dog left ventricle because frequent arrhythmias occurred at such a high load, and high pressure of postextrasystolic potentiated contractions tended to tear away the mitral fixation of the balloon*” (Suga et al., 1986b).

In studies using isolated muscle preparations including papillary muscles and trabeculae, the initial length is commonly set at the optimal length where the preparations can produce the highest active force. Under this condition, reconciliation with the PVA concept of H. Suga becomes challenging due to the appearance of contraction-mode dependency and the resulting indecisiveness in calculating ‘U’. Comparing Figures 6, 7, Hisano and Cooper (1987) may not have reached their conclusion if they had calculated ‘U’ using a lower force-length relation. Their revised conclusions, based on our replication in Figure 6, could have been that the relation between VO_2_ and FLA is *not* independent of the mode of contraction and the relation between VO_2_ and peak force can *not* be fitted with only a single regression line. Likewise, our replication in Figure 7 has provided a convincing explanation to the uncertainty of C. L. Gibbs (Gibbs et al., 1992; Kiriazis et al., 1992) as to why the “*activation heat values obtained using the FLA analysis were slightly higher than that found using the heat:stress data*”. The explanation resides in the calculation of ‘U’ where the end-systolic stress-length relation used is lower than that of isometric contraction. This underestimates ‘U’, which shifts the relation between change of enthalpy and SLA to the left, thereby overestimating activation heat.

### Physiological working range

A direct evidence has been recently provided in a study of the beating mouse heart *in vivo* regarding the physiological working length of sarcomeres (Kobirumaki-Shimozawa et al., 2016). They found that the *in vivo* working range of sarcomere length “*was on the shorter end of the resting distribution*”, averaged to around 1.9 µm during diastole. If the optimal sarcomere length is taken to be 2.3 µm, then the physiological sarcomere length translates to around 0.83 *L*_*o*_, which is within the region of low initial length where contraction mode dependence appears otherwise (Figures 5D, 8A).

**FIGURE 11:**
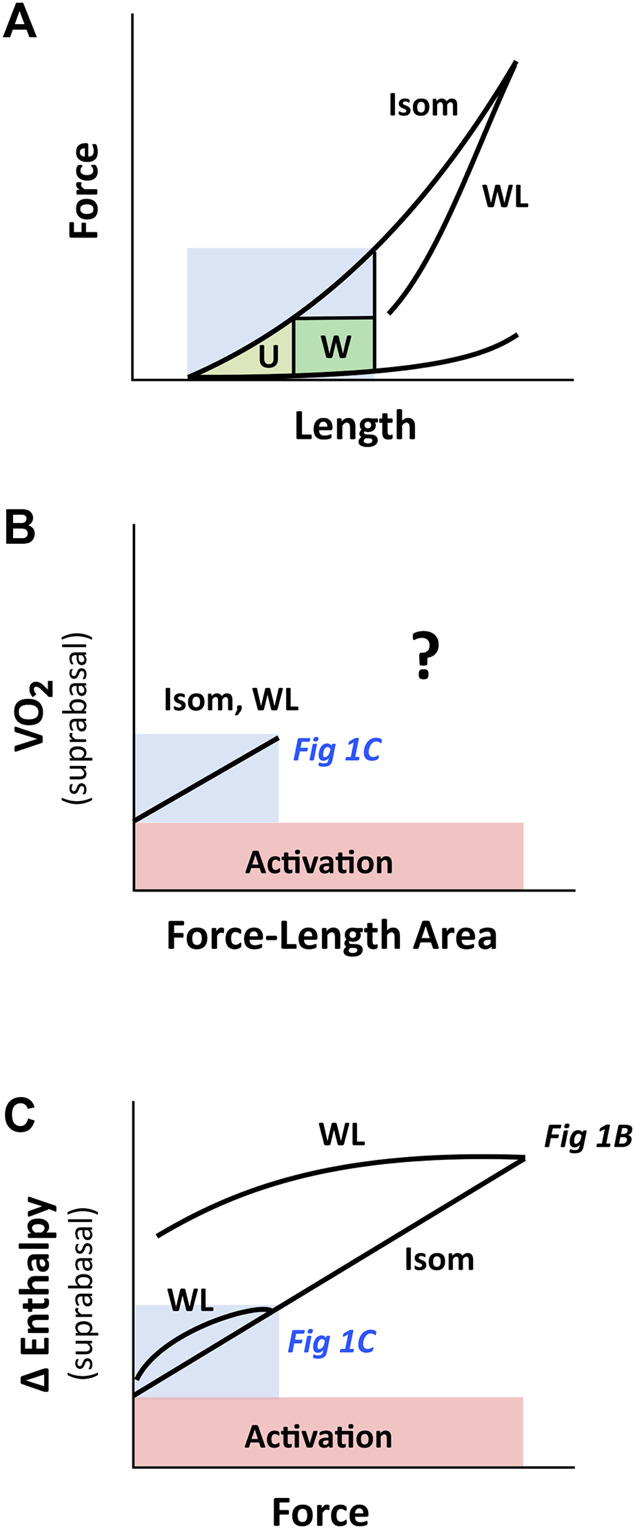
PVA concept of H. Suga on cardiac force-length range. The region coloured in blue indicates the putative physiological working range. **(A)** Cardiac force-length relation is contraction-mode dependent, but may appear otherwise within the region of low initial length, where a single end-systolic force-length relation (the WL relation is very close to the Isom relation) renders the calculation of ‘U’ ambiguous. **(B)** When ‘U’ is calculated from the short-length region (shaded blue; ref Figure 5), the relation between VO_2_ and force-length area appears to be contraction-mode independent (labelled Isom and WL), which yields a single, contraction mode-independent, estimate of activation energy on the *y*-axis. At longer muscle lengths (i.e., beyond the blue region), the relation between VO_2_ and force-length area for WL contractions is uncertain, given the ambiguous estimate of ‘U’ (Figures 6 vs. Figure 7), as indicated by the question mark. **(C)** The relation between change of enthalpy (Δ enthalpy) and force is contraction-mode dependent, where that obtained from work-loop contractions diverges further from isometric contraction the greater the initial muscle length. Thus, at the *y*-intercept, activation energy may appear contraction mode independent when experiments are performed at low initial length. This insight reconciles the inconsistency in the literature regarding activation energy illustrated in Figures 1B,C. The drawing in panel C is based on our experimental findings plotted in Figure 2. The labels ‘Fig 1B’ and ‘Fig 1C’, which are positioned within their respective regions on the Δ enthalpy-force axis, refer to Figures 1B,C, respectively.

Figure 11 illustrates our reconciliation of the mechanoenergetics findings of H. Suga (Figure 1C) with those of C. L. Gibbs (Figure 1B). In essence, the PVA concept of H. Suga applies only at the lower end and within a small range of the force-length relation, which may be in line with the *in vivo* physiological working range of sarcomeres. In isolated heart or isolated muscle studies, experiments are more commonly conducted beyond the physiological range, i.e., at the higher end of the force-length relation. Thus, at high initial lengths, families of lines that denote contraction-mode dependency are obtained on the force-length space and heat-force space.

### Curvilinearity

Under isovolumic or isometric contractions, both methods of estimating activation energy/heat are expected to arrive at the same estimate. This is true only if the calculation of ‘U’ is accurate; and an accurate calculation of ‘U’ requires a reliable measurement of the dead-space volume (*V*_*0*_: volume-axis intercept, where pressure is zero). This requirement is often challenging to meet. In an isolated whole-heart study, this “*could not be done because of the relatively large volume of the balloon wall and cannula placed within the LV*” (Wannenburg et al., 1992). Thus, *V*_*0*_ is often estimated by extrapolation of the isovolumic end-systolic pressure-volume relation (ESPVR) to the volume axis. The problem then focusses on the linearity of the ESPVR. While H. Suga demonstrated a linear ESPVR in the adult canine heart over the small volume range (15 mL–40 mL) within which he typically conducted his experiments, the same volume range as he observed significant nonlinearity of the ESPVR in the heart under baseline, enhanced and depressed contractile states (Suga et al., 1986b). Curvilinear ESPVRs have been reported by several investigators in hearts within the same volume range (15 mL–40 mL) in adult dogs (Ullrich et al., 1954; Burkhoff et al., 1987; Mirsky et al., 1987; Kass et al., 1989). Curvilinear ESPVRs are likewise obtained in hearts of pigs (Davidson et al., 2015), rabbits (Goto et al., 1990), mice (How et al., 2006) and rats (Kissling et al., 1985; Wannenburg et al., 1992; Tachibana et al., 1997; Hata et al., 1998; Misawa et al., 2001; Obata et al., 2018). Polynomial regressions, typically of a quadratic form, have been applied to fit to the curvilinear ESPVR. Alternative forms have been proposed, one of which takes into account myocardial stiffness, several geometric variables and empirically-determined constants (Mirsky et al., 1987), that can fit the ESPVR equally as well as using the quadratic regression but result in different estimates of *V*_*o*_ (Kass et al., 1989). These non-linear fitting models attempt to obtain positive values of *V*_*o*_ particularly when a linear regression would otherwise result in an extrapolation to the negative side of the volume-axis yielding physiologically nonsensical estimates of *V*_*o*_ (Kass et al., 1989; Tachibana et al., 1997). Given that both *V*_*o*_ and ‘U’ are susceptible to the fitting regression models, we stress here that their unfitted estimates can lead to spurious results for activation energy (Wannenburg et al., 1992). To preclude the complications associated with estimating *V*_*o*_ and ‘U’, we encourage that VO_2_ be plotted against isovolumic systolic pressure (P) which can be directly measured experimentally, rather than against the derived PVA. This is in line with plotting heat against measured isometric force, in the search for an accurate estimate of activation heat. Indeed, VO_2_ has been shown to correlate with isovolumic systolic pressure (Monroe and French, 1961; Krukenkamp et al., 1986) or with isometric tension (Gunning et al., 1973; Cooper et al., 1974), where the *y*-intercept provides an estimate of activation energy.

Curvilinearity has likewise appeared in the relation between VO_2_ and PVA under isovolumic contractions (Wannenburg et al., 1992; Sakata et al., 2006) and ejecting contractions (Nozawa et al., 1989; Nozawa et al., 1994); i.e., the data points relating VO_2_ and PVA were fitted with linear regression even though they appear to be better fitted by quadratic regression. We observed the same feature in the plot between change of enthalpy and FLA obtained from an isolated papillary muscle (Gibbs et al., 1992). In the present study, we show that the relation between change of enthalpy and SLA under isometric contractions is curvilinear (Figure 4). The underlying basis for this curvilinearity is that twitch duration is not zero at the sarcomere length that reduces force to zero, as uncovered in our previous mathematical modelling study (Han et al., 2012c). Over the whole spectrum of muscle length, i.e., from minimal to optimal, which can be achieved using isolated muscle preparations, isometric contractions produce a curvilinear change of enthalpy-SLA relation. This means that over a smaller, confined, range of muscle length, the curvilinear relation between change of enthalpy and SLA can appear linear, and would result in a higher estimate of activation heat. Thus, we caution against the interpretation of activation heat obtained from studies confined to smaller range and longer muscle lengths, where there is lack of data points close to the *y*-intercept (Matsushita et al., 1995; Takeuchi et al., 2001; Ramanathan et al., 2002).

In conclusion, the present study has resolved the inconsistency among studies in the estimation of cardiac activation energy. The range of muscle length (ventricular volume) at which experiments were conducted contributes to the inconsistency. Low muscle lengths render the cardiac force-length relation contraction mode independent and, hence, yield a contraction mode-independent estimate of activation energy. In contrast, at longer muscle lengths, particularly in experiments using isolated muscle preparations at optimal length, contraction mode-dependence of cardiac force-length relation is evident, and activation energy can be estimated accurately only from isometric contractions. The higher *y*-intercept extrapolated from work-loop contractions contains heat of shortening in addition to activation heat. In addition, caution is needed when using the pressure-volume area concept in estimating activation energy as it necessitates an apparent geometrical calculation of ‘U’ area, which becomes ambiguous when contraction mode-dependence produces more than one cardiac pressure-volume (force-length) relation.

## Data Availability

The raw data supporting the conclusions of this article will be made available by the authors, without undue reservation.
