# Erratum: Resolving an inconsistency in the estimation of the energy for excitation of cardiac muscle contraction

**DOI:** 10.3389/fphys.2023.1328389

**Published:** 2023-11-03

**Authors:** Frontiers Production Office

**Affiliations:** Frontiers Media SA, Lausanne, Switzerland

**Keywords:** cardiac energetics, activation heat, pressure-volume area, force-length work, calcium handling, end-systolic

Due to a production error, the **Article title** was not updated to “*Resolving an inconsistency in the estimation of the energy for excitation of cardiac muscle contraction*.”

Due to the lack of necessary copyright permissions, Figures 6, 10 have been replaced with updated Figures and their captions have been updated. The correct figures and captions appear below.

**FIGURE 6:**
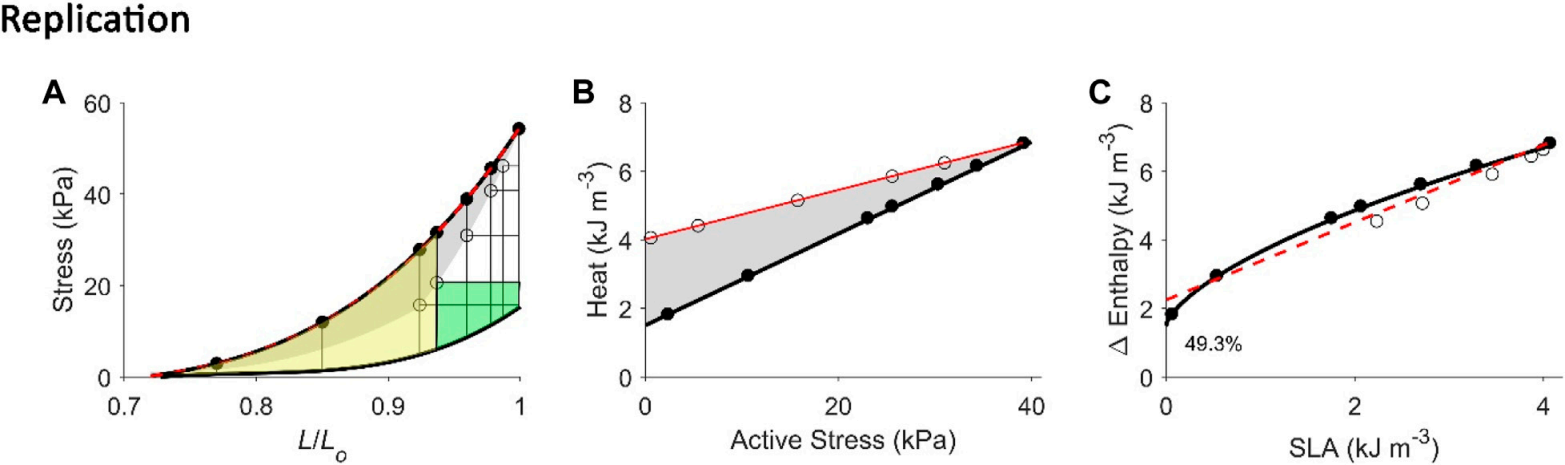
Replicating mechanoenergetics results where work-loop contractions are performed at optimal length. Our replication of Figure 4 of Hisano and Cooper (1987). Their experiments were conducted on an isolated papillary muscle undergoing isometric contractions at various lengths and afterloaded work-loop contractions at the optimal length. Four of seven isometric contractions coincide with the end-systolic length of the four lower work loops. A selected work loop is coloured green to indicate muscle work output; the adjacent area that was taken by the authors as “U” is coloured yellow (same colour convention as in Figure 1A). Note that for this selected work loop, its end-systolic point is lower than that achieved by the isometric contraction at the matched end-systolic length. The authors fitted a linear line to illustrate contraction mode independence of the relation between myocardial oxygen consumption and peak force, as well as with force-length area. **(A)** Our replication of their results, consisting of five afterloaded work loops at the initial length of Lo (end-systolic points indicated by open circles) and seven isometric contractions (end-systolic points indicated by filled circles) where four of them coincide with the end-systolic length of the four lower work loops. The selected work loop is coloured to illustrate areas representing work (green) and “U” (yellow). The end-systolic line (broken red line) is fitted only to the isometric contractions, as is consistent with their approach. **(B)** Equivalent heat-stress points of the replicated end-systolic points in **(A)**. The red line is fitted to the work-loop heat-stress points (open circles) that sit higher than the isometric heat-stress points (filled circles) for a given stress, which resembles their data above. This case is also obtained in our experiments, as graphed in Figure 2B. **(C)** The resultant Δ Enthalpy-SLA points are fitted with linear regression (broken red line) regardless of the mode of contraction. The resultant *y*-intercept of the linear relation is 49.3% higher than the *y*-intercept of the isometric relation (solid line; transcribed from Figure 4C). Two reassuring details of our replication are that the work loop at the lowest afterload encompasses the end-systolic lengths of the work loops at the four higher afterloads (panel A), and the work-loop points sit higher than the isometric points on the heat-stress plane (panel B).

**FIGURE 10:**
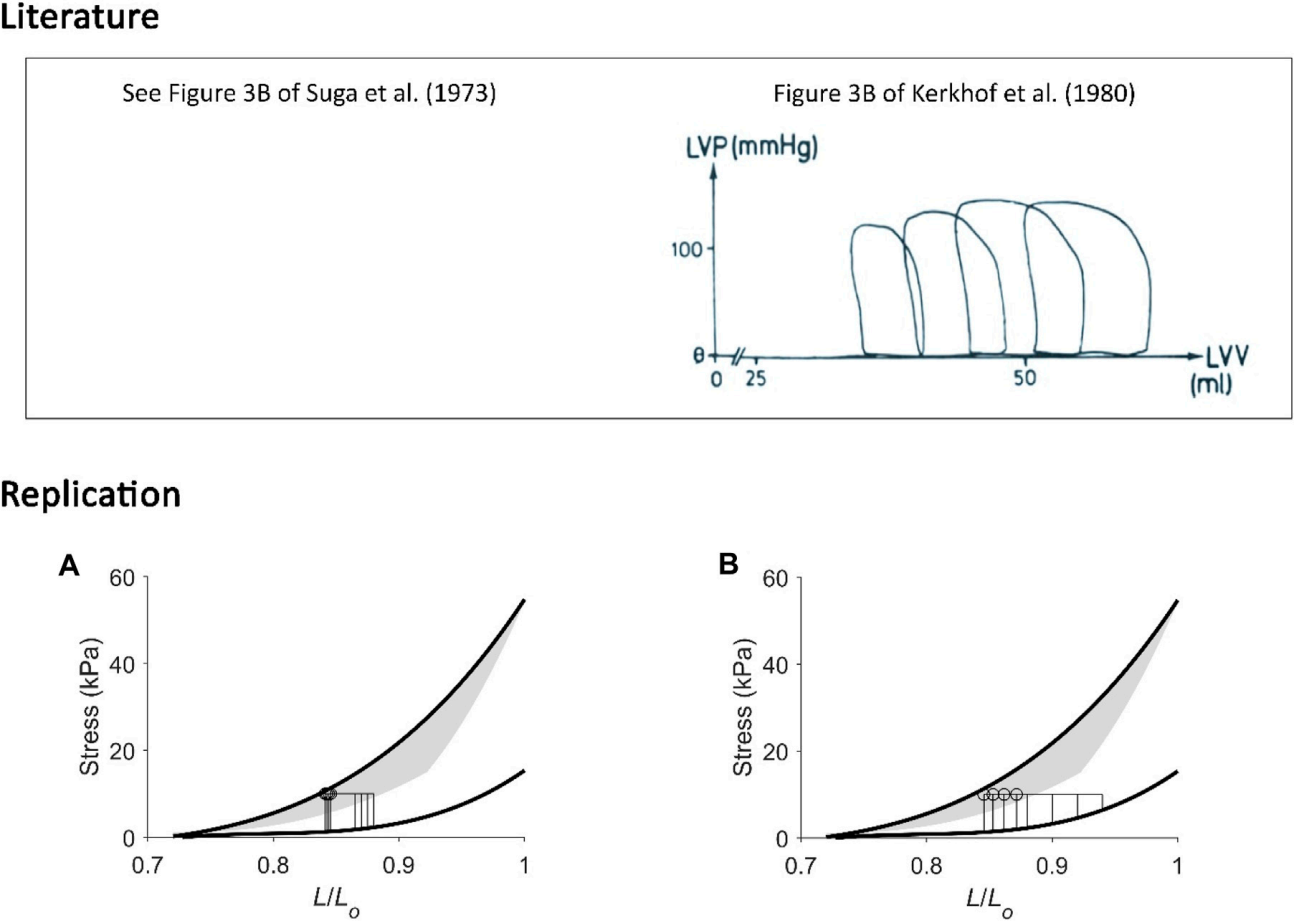
Replicating pressure-volume loops at low and at high initial volumes. **(A)** Our replication of Figure 3B of Suga et al. (1973). **(B)** Our replication of Figure 3B of Kerkhof et al. (1980), the figure of which is reproduced above in the box labelled “Literature” with permission from Springer Nature via Copyright Clearance Center. In these two unrelated experiments, isolated canine hearts were subjected to four pressure-volume loops. The mean arterial (aortic) pressure was kept constant while initial volume was varied within a lower range (left-hand panel) or a wider range at higher initial volumes (right-hand panel). In the former case **(A)**, end-systolic points from the four work loops were close to one another; they aligned close to the isovolumic pressure-volume relation, and they all share a similar “U.” In the latter case **(B)**, end-systolic points are noticeably different to one another, and our replication shows that they diverge from the isometric stress-length relation, where the greater the initial length, the further away the end-systolic point. The calculation of “U” in the latter case is ambiguous, i.e., could be by either using four different work-loop end-systolic stress-length relations or using the isometric stress-length relation with the prevailing initial length at each of the four end-systolic lengths of each work loop (as inspired from Figure 6).

The publisher apologizes for this mistake. The original version of this article has been updated.

